# Iowa

**Published:** 1896-10

**Authors:** 


*Iowa*. Hog-cholera is said to prevail to a considerable extent at Cerro Gordo.

